# Statins and Antimicrobial Effects: Simvastatin as a Potential Drug against *Staphylococcus aureus* Biofilm

**DOI:** 10.1371/journal.pone.0128098

**Published:** 2015-05-28

**Authors:** Talita Signoreti Graziano, Maria Claudia Cuzzullin, Gilson Cesar Franco, Humberto Osvaldo Schwartz-Filho, Eduardo Dias de Andrade, Francisco Carlos Groppo, Karina Cogo-Müller

**Affiliations:** 1 Pharmacology, Anesthesiology and Therapeutics, Department of Physiological Sciences, Piracicaba Dental School, State University of Campinas, Piracicaba, São Paulo, Brazil; 2 Department of General Biology, Area of Physiology and Pathophysiology, State University of Ponta Grossa, Paraná, Brazil; 3 Department of Dentistry, Implantology Area, University of Santo Amaro, São Paulo, São Paulo, Brazil; Ghent University, BELGIUM

## Abstract

Statins are important lipid-lowering agents with other pleiotropic effects. Several studies have explored a possible protective effect of statins to reduce the morbidity and mortality of many infectious diseases. *Staphylococcus aureus* is one of the main pathogens implicated in nosocomial infections; its ability to form biofilms makes treatment difficult. The present study observed the MIC of atorvastatin, pravastatin and simvastatin against *S*. *aureus*, *Pseudomonas aeruginosa*, *Escherichia coli* and *Enterococcus faecalis*. Simvastatin was the only agent with activity against clinical isolates and reference strains of methicilin-sensitive *S*. *aureus* (MSSA) and methicillin-resistant *S*. *aureus* (MRSA). Thus, the effects of simvastatin on the growth, viability and biofilm formation of *S*. *aureus* were tested. In addition, a possible synergistic effect between simvastatin and vancomycin was evaluated. Simvastatin’s MIC was 15.65 µg/mL for *S*. *aureus* 29213 and 31.25 µg/mL for the other strains of *S*. *aureus*. The effect of simvastatin was bactericidal at 4xMIC and bacteriostatic at the MIC concentration. No synergistic effect was found between simvastatin and vancomycin. However, the results obtained against *S*. *aureus* biofilms showed that, in addition to inhibiting adhesion and biofilm formation at concentrations from 1/16xMIC to 4xMIC, simvastatin was also able to act against mature biofilms, reducing cell viability and extra-polysaccharide production. In conclusion, simvastatin showed pronounced antimicrobial activity against *S*. *aureus* biofilms, reducing their formation and viability.

## Introduction

Simvastatin is a lipophilic drug that belongs to the group of statins. The statins are lipid-lowering agents that are involved in the reduction of cardiovascular morbidity and mortality [1]. These drugs exhibit a good margin of safety and tolerability with a low frequency of side effects and are the most commonly used agents for the reduction of lipids in patients with elevated cholesterol levels [2, 3]. All statins act by the same mechanism of action, competitively inhibiting the enzyme 3-hydroxy-3-methyl-glutaryl-CoenzymeA reductase (HMG-CoA) and causing a decrease in the biosynthesis of cholesterol and increased removal of circulating low-density lipoprotein (LDL) [4, 5].

Statins have effects other than lipid reduction, called pleiotropic effects, such as anti-inflammatory and immunomodulatory activities [6–8]. Many studies have evaluated the effect of statins on the prevention, morbidity and mortality of various infectious diseases. Some of these studies have shown that statins can prevent the establishment of infections or even reduce mortality rates in patients routinely taking statins. In patients with bacteremia and sepsis, the use of statins was associated with lower mortality in recent studies [9–13]. However, other studies did not find the same protective effect [14, 15]. Interestingly, some studies have demonstrated an antimicrobial potential for statins against different bacterial species [16–20]. For example, simvastatin was able to inhibit host-cell invasion [17] and *Staphylococcus aureus* growth [19, 21, 22]. In addition, atorvastatin, simvastatin and rosuvastatin showed activity against several reference bacteria and clinical isolates [19, 22].

*Pseudomonas aeruginosa*, *Staphylococcus epidermidis*, *Enterobacter* spp, *Escherichia coli*, *Klebsiella* spp, *Acinetobacter* spp and especially *Staphylococcus aureus* are frequently involved in nosocomial infections [23]. *S*. *aureus*, one of the most important etiological agents of both nosocomial and community-onset infections, produces several virulence factors, such as toxins, adhesins and components of immune evasion [24]. These characteristics along with the ability to form biofilms results in an increased capability of surviving in a hostile environment [25]. The cells attached to the biofilm produce an extracellular matrix composed of polysaccharides, proteins and DNA [26]. This matrix allows the bacterial cell to protect itself against the host defense system and antimicrobial agents, which makes the treatment of nosocomial infections difficult [26].

In the present study, the activity of simvastatin, atorvastatin and pravastatin was evaluated against a range of clinically important pathogens through susceptibility methods. A possible synergistic action of vancomycin and simvastatin was also tested using a microtiter checkerboard method. Finally, the effects of simvastatin on *S*. *aureus* adhesion, biofilm viability and polysaccharide and protein production were further evaluated.

## Materials and Methods

### Chemicals and Experimental Groups

Atorvastatin (atorvastatin calcium salt trihydrate), pravastatin (pravastatin sodium salt hydrate) and simvastatin (Sigma Chemical Co—St. Louis, MO, USA) were used for minimum inhibitory concentration (MIC) experiments. Atorvastatin and simvastatin were dissolved in 100% DMSO, and pravastatin was dissolved in distilled-deionized water. The final concentration of DMSO was 2.5%. Both gentamicin and vancomycin dissolved in deionized water were used as antimicrobial standards.

Formulations were distributed into microtiter plate wells as follows: a) experimental groups (culture medium + bacteria + statin or antimicrobial standard); b) positive control (culture medium + bacteria); c) vehicle control (culture medium + bacteria + DMSO); d) negative control (culture medium + statin or antimicrobial standard); e) medium negative control (culture medium); f) negative vehicle control (culture medium + DMSO). All tests were performed with six replicates on at least two separate occasions.

### Bacterial strains and Culture Conditions

The following reference strains were used: *Staphylococcus aureus* ATCC 29213 (methicilin-sensitive *S*. *aureus*—MSSA), *S*. *aureus* ATCC 6538 (a standard for testing antimicrobial agents), *S*. *aureus* ATCC 14458 (enterotoxin B gene present), *S*. *aureus* ATCC 33591 (methicillin-resistant *S*. *aureus*—MRSA), methicillin-oxacillin-resistant *S*. *aureus* ATCC 43300 (MRSA; mecA gene present), *Pseudomonas aeruginosa* ATCC 27853, *P*. *aeruginosa* ATCC 25619, *Escherichia coli* ATCC 25922, *E*. *coli* ATCC 10536, *Enterococcus faecalis* ATCC 29212. *S*. *aureus* isolated from clinical samples were also tested. MRSA isolated from sputum samples (strains HC 3817719, HC 10106876, HC 9120358) and MSSA from blood cultures (strains HC 12092392 and HC 985444) were kindly provided by Professor Carlos Emilio Levy (Faculty of Medical Sciences, Department of Clinical Pathology, State University of Campinas, Brazil). All bacteria were stored in Tryptic Soy broth (TSB—Difco Co., Detroit, MI, USA) with 20% glycerol at -80°C. Strains were routinely cultured on TSA plates, in aerobic conditions, at 35°C.

Mueller Hinton broth (MHB—Difco Co.) was used for MIC and planktonic tests. For biofilm experiments, *S*. *aureus* ATCC 29213, HC 3817719 and HC 12092392 were cultivated in Brain Heart Infusion (BHI—Difco Co., Detroit, MI, USA) with 1% D-glucose (Sigma Chemical Co—Poole, UK). These strains were chosen because they displayed a better biofilm formation profile in tests performed in our laboratory (data not shown).

For all of the following tests, the bacterial inoculum was prepared in 0.9% NaCl at an optical density of 0.1 at 660 nm, which was equivalent to 1 to 2 x 10^8^ CFU/mL. In each test, the amount of the initial bacterial load was 5 x 10^5^ CFU/mL.

### Minimum Inhibitory Concentration (MIC)

The MIC was determined by the broth microdilution method as previously described by The Clinical and Laboratory Standards Institute [27]. Concentrations for all statins ranged from 250 to 0.24 μg/mL and from 100 to 0.06 μg/mL for antimicrobial standards. Two-fold dilutions were obtained in 96-well plates with 100 μL of MHB per well. Then, the bacterial suspension (100 μL) was inoculated, and the plates were incubated for 24 h at 35°C. The lowest concentration with any visible bacterial growth was taken as the MIC. In addition, bacterial growth was assessed by optical density measurement (660 nm).

### Time-kill assays

For all of the following assays, vancomycin and simvastatin (the statin with the best antimicrobial activity) were tested against *S*. *aureus* ATCC 29213.

The time-kill assay was adapted from a previously described method [28, 29]. MIC and 4xMIC concentrations were chosen according to the data obtained from the previous experiments. The time-kill assay was performed as described in the MIC assay, and 25-μL samples were taken from the microtiter plates after 0, 2, 4, 8 and 12 h of incubation and spread on TSA plates. Viable colonies were counted after 24 h incubation. Killing curves were constructed by plotting the log_10_ CFU/mL versus time over 12 h.

### Post-antibiotic effect (PAE)

Determination of the PAE was adapted from previously described methods [28, 30]. Concentrations were four times higher than the MIC (4xMIC), two times higher than the MIC (2xMIC), the MIC and one-half the MIC (½xMIC). Twenty-four-well plates containing the testing substances were incubated for 2 h. Samples were centrifuged for 5 minutes at 1400 *g*, the supernatant was removed, and new fresh medium was added. This procedure was repeated two times to ensure complete removal of drugs.

The pellet was suspended in culture medium, diluted 1:10 in tubes containing a final volume of 5 mL of culture medium and incubated at 35°C. Samples of 25 μL were collected before and after washing every hour until there was visible growth (OD_660nm_ = 0.3) and plated on TSA plates to obtain viable counts. The PAE was calculated using the following equation: PAE = T—C, where T is the time required for the initial bacterial culture to increase by 1 log_10_ CFU/mL from the removal of the antimicrobial, and C represents the time required for bacterial cultures not treated with an antimicrobial to increase 1 log_10_ CFU/mL.

### Checkerboard Microdilution Assay

To evaluate a possible interaction between simvastatin and vancomycin, a checkerboard microdilution assay was used [31, 32]. Simvastatin and vancomycin were prepared at four times of the final concentration in separated plates. Then, 50 μL simvastatin and 50 μL of vancomycin were mixed and transferred to a new plate. Finally, 100 μL of bacterial suspension was inoculated, and the plates were incubated. Analyses of results were based on the value of the FICI (fractional inhibitory concentration index) that was calculated using the following formula:

$$\sum \;\text{FICI}\;=\;{\text{FICI}}_{\text{A}}\;+\;{\text{FICI}}_{\text{B}}={\text{MIC}}_{\text{AB}}\;/\;{\text{MIC}}_{\text{A}}\;+\;{\text{MIC}}_{\text{BA}}\;/\;{\text{MIC}}_{\text{B}}$$

where MIC_A_ and MIC_B_ are the MICs of drugs A and B when acting alone and MIC_AB_ and MIC_BA_ are the MICs of drugs A and B when acting in combination, respectively.

FICI values < 0.5 represent synergism in the interaction between drugs. FICI values between 0.5 < FICI < 4.0 are classified as indifferent, and FICI values > 4.0 are classified as antagonism.

### Biofilm formation assay

For this assay, vancomycin and simvastatin were tested against *S*. *aureus* ATCC 29213, MRSA (HC 3817719) and MSSA (HC 12092392).Biofilm formation experiments were conducted using U-bottom, 96-well plates. Concentrations of simvastatin and vancomycin ranged from ^1^/_28_xMIC to 4xMIC. Two-fold dilutions were obtained in 96-well plates with 100 μL of BHI with 1% glucose per well. Then, the bacterial suspension (100 μl) was inoculated, and the plates were incubated for 24 h at 35°C.

After 24 h of incubation, the plates were washed with distilled-deionized water to remove dead or unattached cells. After drying at room temperature, the quantification of biofilm formed in each well was made by optical density measurement (OD_575nm_) after the addition of 0.4% crystal violet solution and 100% ethanol [33].

### Analysis of biofilm formation by scanning electron microscopy (SEM)

The effect of simvastatin on biofilm formation was analyzed by SEM. The biofilm was formed as described in the previous section with the following modifications. After growing for 24 h in Lab-Tek Chambers (Nunc, Naperville, IL, USA), 3% glutaraldehyde/PBS (v / v, pH 7.4) was added for 12 h. Next, the samples were dehydrated with ethanol (50% to 100%). The biofilms were coated with gold and examined using a JEOL JSM5600LV (JEOL Ltd., Tokyo, Japan) scanning electron microscope [34].

### Biofilm viability assay

To assess the effect of simvastatin on the viability of mature biofilms, 24-h biofilms were exposed to the antimicrobial drugs. Biofilms of *S*. *aureus* ATCC 29213 were formed in cellulose acetate membranes filters (diameter: 25 mm; pore size: 0.2 μm; Sartorius AG, Germany) placed at the bottom of 6-well plates.

After biofilm formation, membranes were transferred to a new plate containing culture media and the antimicrobial substances at 4xMIC. The plates were then incubated for 24 h at 35°C. Membranes were removed from the plates and washed three times with 5 mL of 0.9% NaCl for 5 s each. Then, membranes were transferred to polystyrene tubes containing 5 mL of 0.9% NaCl and sonicated (Vibra Cell 400W, Sonics & Materials Inc., Newtown, CT, USA) in the 5% range and 20 W for 30 seconds for dispersion of the biofilm. Samples of 10 μL were collected, plated on TSA and kept at 35°C for 24 h. After this period, viable counts were performed.

### Quantification of polysaccharides, proteins and biomass

In addition to viability of the biofilm, the production of polysaccharides, proteins and biomass (dry weight) after exposure to simvastatin were also analyzed.

The biofilms were formed and sonicated as described in the biofilm viability assayA portion (800 μL) of the biofilm suspension was collected, and the extraction of insoluble extracellular polysaccharides was performed with 1 M NaOH as described by Aires et al. [35]. After this step, biofilms were boiled at 100°C for 15 minutes for cell disruption, allowing extraction of intracellular polysaccharides with 1 M NaOH [35]. Quantification was performed using the phenol-sulfuric method [36]. Protein extraction was conducted using a 2 M NaOH solution [37] and subsequently quantified by colorimetric assays through BCA protein quantification (Thermo Scientific, Scottsdale, AZ, USA). For biomass quantification (dry weight), 1000 μL of each sample was centrifuged, and the pellet was dried in a lyophilizer (Lyo Chamber Guard Christ LCG 121505 PMMA (Nova Analítica) Alpha 2–4 LD plus) and then weighed.

### Statistics

All tests were performed using six replicates per group on at least two separate occasions. Data were analyzed by using GraphPad version 5.00 (San Diego, CA, USA). Data distribution was tested using the Shapiro-Wilks test. Data showing a normal distribution were compared using an ANOVA, and differences between control and treatment groups were determined using the Bonferroni *post-hoc* test. Non-normally distributed data were analyzed by Kruskal-Wallis and Dunn *post-hoc* tests. The significance level was set at 5%.

## Results

### Only simvastatin has antibacterial activity against *S*. *aureus*

Simvastatin showed activity against all strains of *S*. *aureus*, but had no effect against the other species tested. The MIC values for simvastatin and the antimicrobial standards are shown in Table 1. No MIC for atorvastatin and pravastatin were found at the concentrations tested. The concentrations of DMSO (2.5% V/V) used in all tests did not interfere with bacterial growth. *P*. *aeruginosa* 27853 and 25619, *E*. *coli* 25922 and 10536 were resistant to vancomycin, but only *S*. *aureus* 43300 was resistant to gentamicin. All MIC values for the antibiotics were in accordance with CLSI.

**Table 1:** MIC (μg/mL) for simvastatin, gentamicin and vancomycin.

Bacterial strains	Simvastatin	Gentamicin	Vancomycin
***S*. *aureus* 29213**	15.65	0.78	1.56
***S*. *aureus* 33591**	31.25	3.12	1.56
***S*. *aureus* 14458**	31.25	0.78	1.56
***S*. *aureus* 43300**	31.25	——	1.56
***S*. *aureus* 6538**	31.25	1.56	1.56
***S*. *aureus* HC 3817719**	31.25	0.78	1.56
***S*. *aureus* HC 10106876**	31.25	1.56	1.56
***S*. *aureus* HC 9120358**	31.25	1.56	3.12
***S*. *aureus* HC 12092392**	31.25	0.78	1.56
***S*. *aureus* HC 985444**	31.25	0.78	1.56
***P*. *aeruginosa* 27853**	——	0.78	——
***P*. *aeruginosa* 25619**	——	0.39	——
***E*. *coli* 25922**	——	1.56	——
***E*. *coli* 10536**	——	0.78	——
***E*. *faecalis* 29212**	——	6.25	3.12

**Fig 1:**
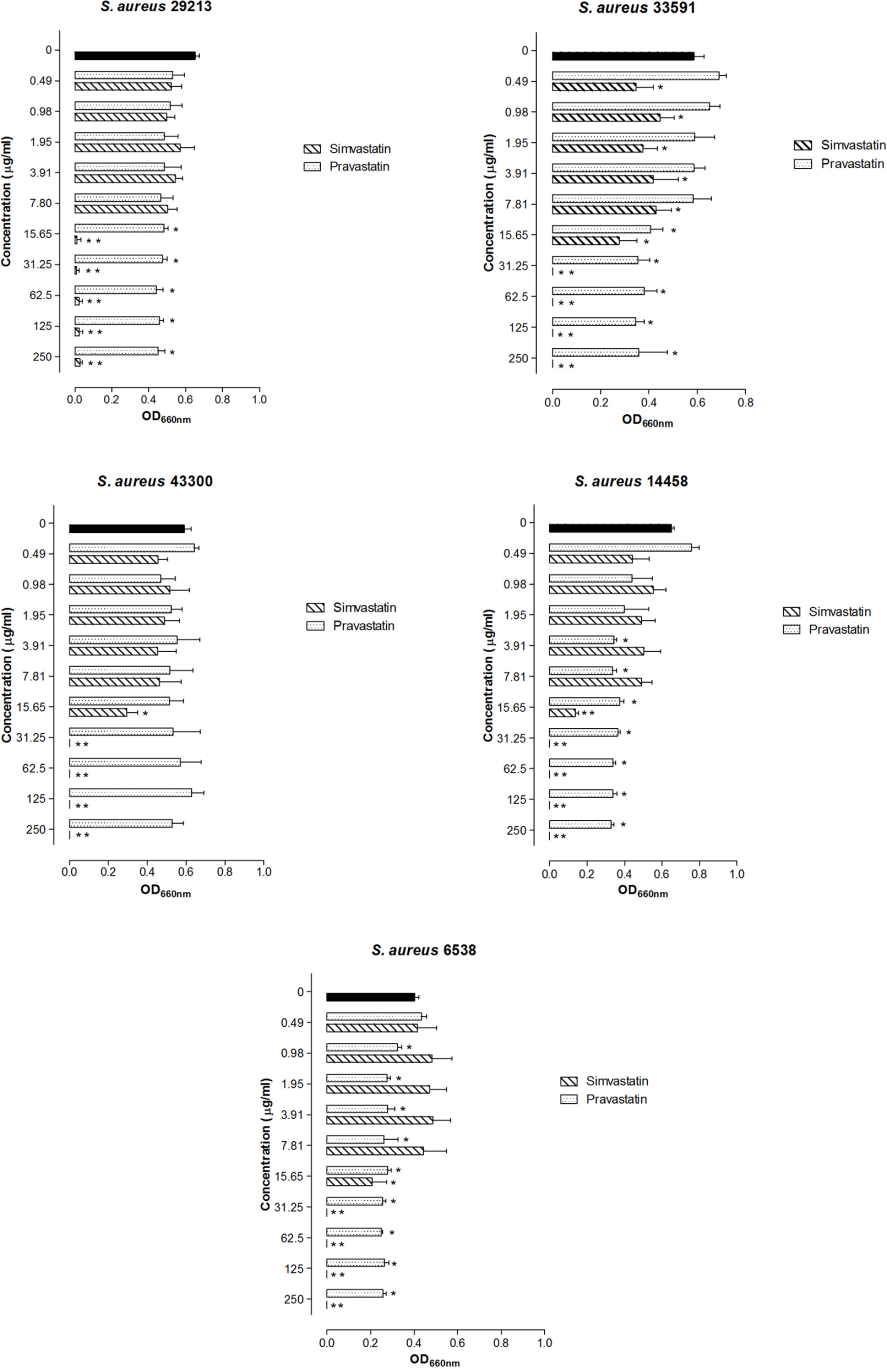
Mean and standard deviation of optical density (660 nm) representing the bacterial growth of *S*. *aureus* when exposed to simvastatin and pravastatin for 24 h. Significant differences between the treatment and the control group were considered when *p<0.05, **p<0.01 (ANOVA, Bonferroni).

The effect of statins on *S*. *aureus* strains is dose and drug dependent, as observed in Fig 1. Pravastatin also caused a reduction in the growth of *S*. *aureus* strains, but it did not completely inhibit those strains. Simvastatin effects against *S*. *aureus* were more prominent, even with sub-MIC concentrations (p < 0.05, ANOVA-Bonferroni).

**Fig 2:**
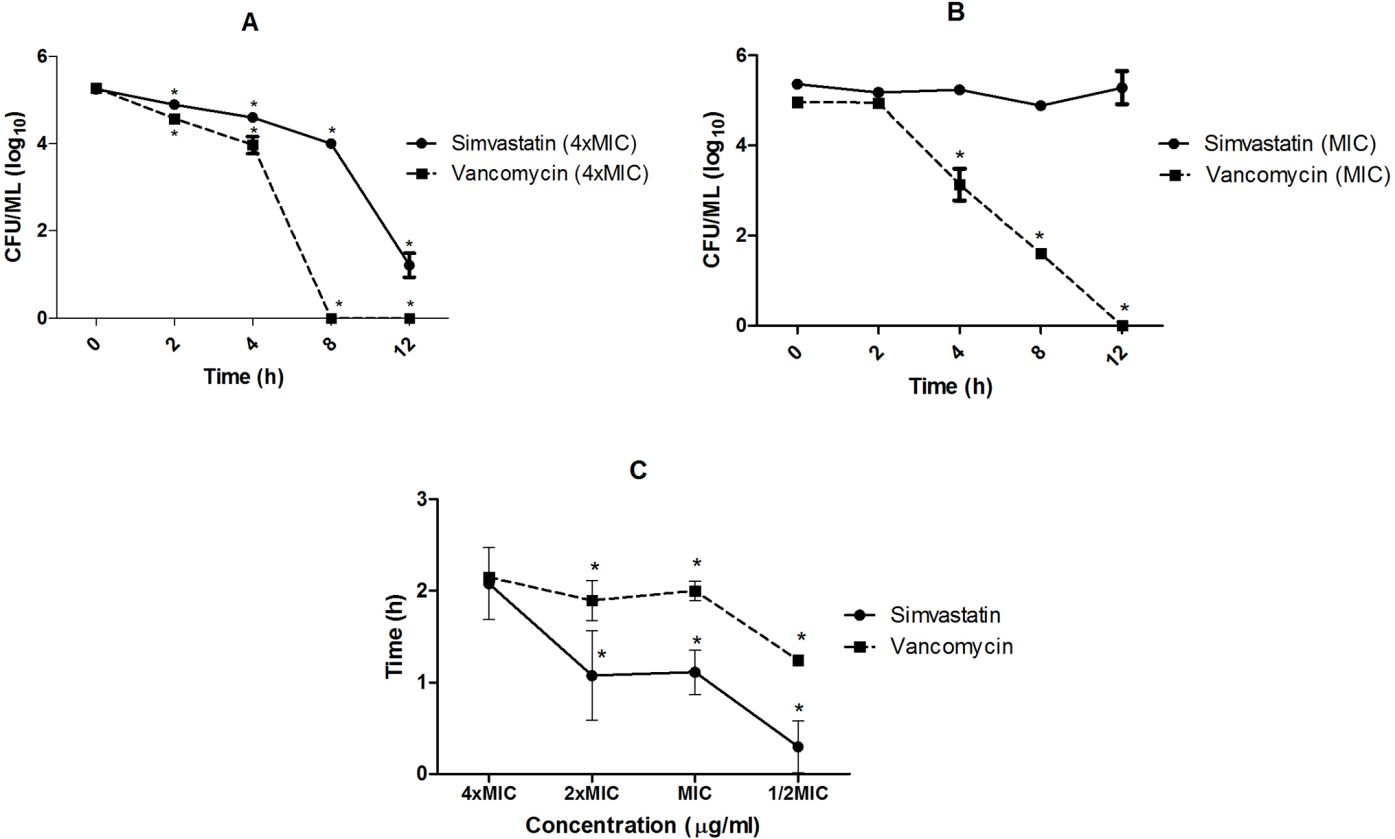
A. Effect of simvastatin and vancomycin on cell viability during 12 h exposure, concentration equivalent to 4xMIC. B. Effect of simvastatin and vancomycin on cell viability during 12 h exposure, concentration equivalent to MIC. Comparisons were made among the different times of exposure and the time 0, before exposure to the substances (control). C. Post-antibiotic effect (PAE) of simvastatin and vancomycin. PAE were compared at each concentration between the substances. (*p<0.05; ANOVA-Bonferroni).

Fig 2 shows the effect of simvastatin on *S*. *aureus* cell viability during 12 h of exposure. Simvastatin at a concentration of 4xMIC exhibited a bactericidal effect against *S*. *aureus* ATCC 29213 and caused a significant reduction in the number of viable cells (Fig 2A), but the MIC concentration showed a bacteriostatic effect (Fig 2B) because the number of cells remained constant during 12 h of exposure to this substance. Vancomycin showed a bactericidal effect for both concentrations tested. However, more time was required to kill 100% of cells at the MIC.

Fig 2C shows the post-antibiotic effect (PAE) for both drugs. Vancomycin showed a greater PAE than simvastatin; however, no differences (p > 0.05) between the PAEs of the two drugs were observed at 4xMIC. DMSO did not have any effect in either time-kill or PAE assays.

### Simvastatin has no synergistic effect with Vancomycin

A combination of simvastatin and vancomycin was tested to evaluate if there was any interaction between the two antimicrobials. FICI values lower than 0.5 represent synergism, values between 0.5 and 4.0 are classified as indifferent and FICI values higher than 4.0 are classified as antagonism. In the checkerboard test, FICI values found were higher than 0.5 as shown in Table 2, indicating that there is no synergistic or antagonist effect between simvastatin and vancomycin against *S*. *aureus*.

**Table 2:** FICI values for association between simvastatin and vancomycin.

Bacterial strain	FICI
***S*. *aureus* 29213**	0.56
***S*. *aureus* 33591**	1.06
***S*. *aureus* 14458**	1.00
***S*. *aureus* 43300**	1.06
***S*. *aureus* 6538**	1.03

### Simvastatin inhibits biofilm formation of *S*. *aureus*

**Fig 3:**
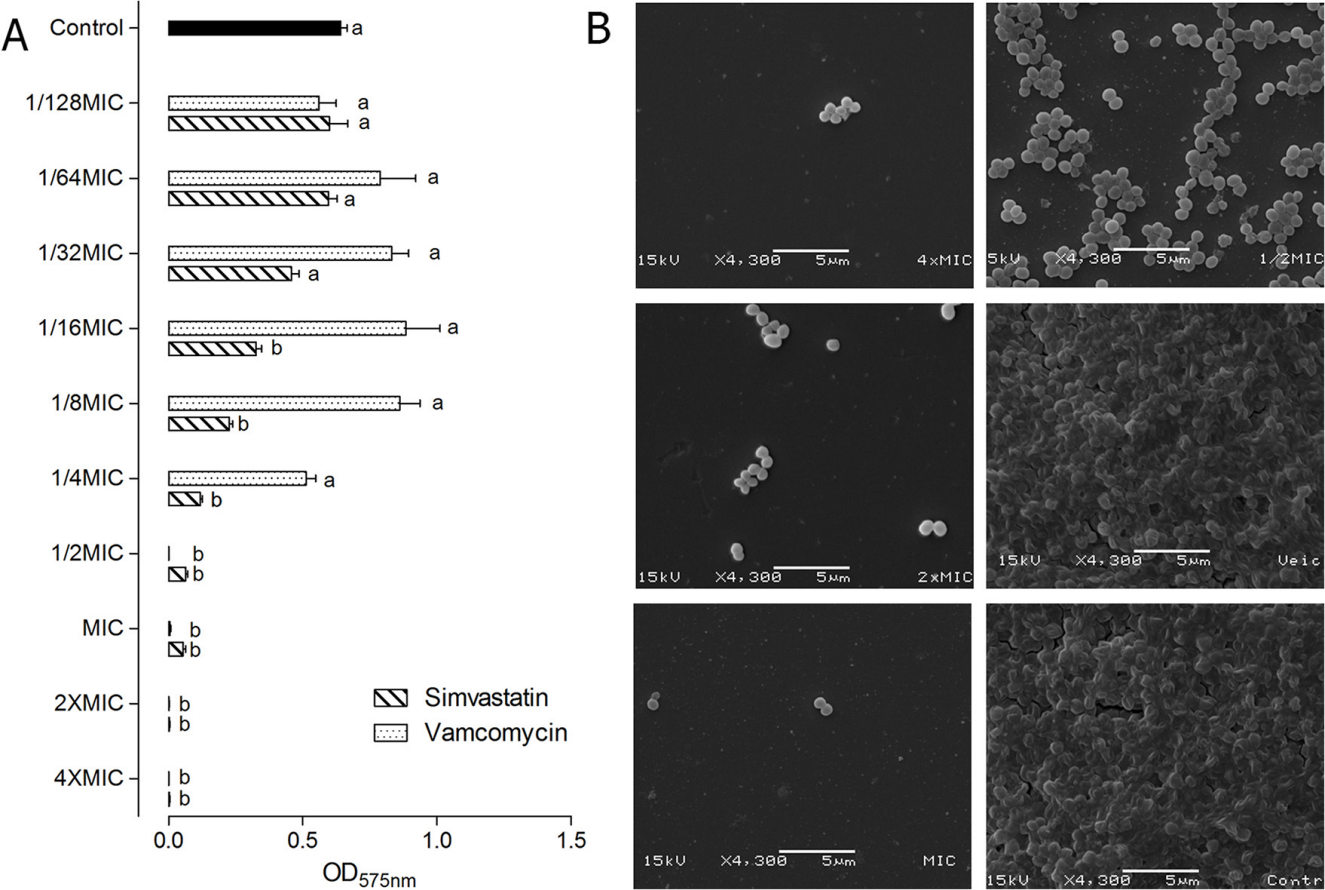
A. Mean and standard deviation of optical densities (575 nm) representing the biofilm formation of *S*. *aureus* 29213 in the presence of simvastatin and vancomycin. Different letters represent significant differences; comparisons were made for each dilution, the control group and each concentration of simvastatin and vancomycin. B. Images obtained by SEM showing biofilm formation of *S*. *aureus* 29213 in the presence of simvastatin. The 4xMIC, 2xMIC and MIC concentrations are in column 1 (top to bottom). The ½xMIC concentration, vehicle group (DMSO) and control group are in column 2 (top to bottom). (p<0.05, two-way ANOVA, Bonferroni).

Fig 3A shows the optical densities for simvastatin and vancomycin cultures representing the effects of these substances on biofilm formation by *S*. *aureus*. Substances were added at the beginning of the biofilm formation assay. The concentrations tested ranged from 1/128xMIC to 4xMIC, and thus, the concentrations for each drug were: 0.12–62.6 μg/mL for simvastatin and 0.012–6.24 μg/mL for vancomycin.

**Fig 4:**
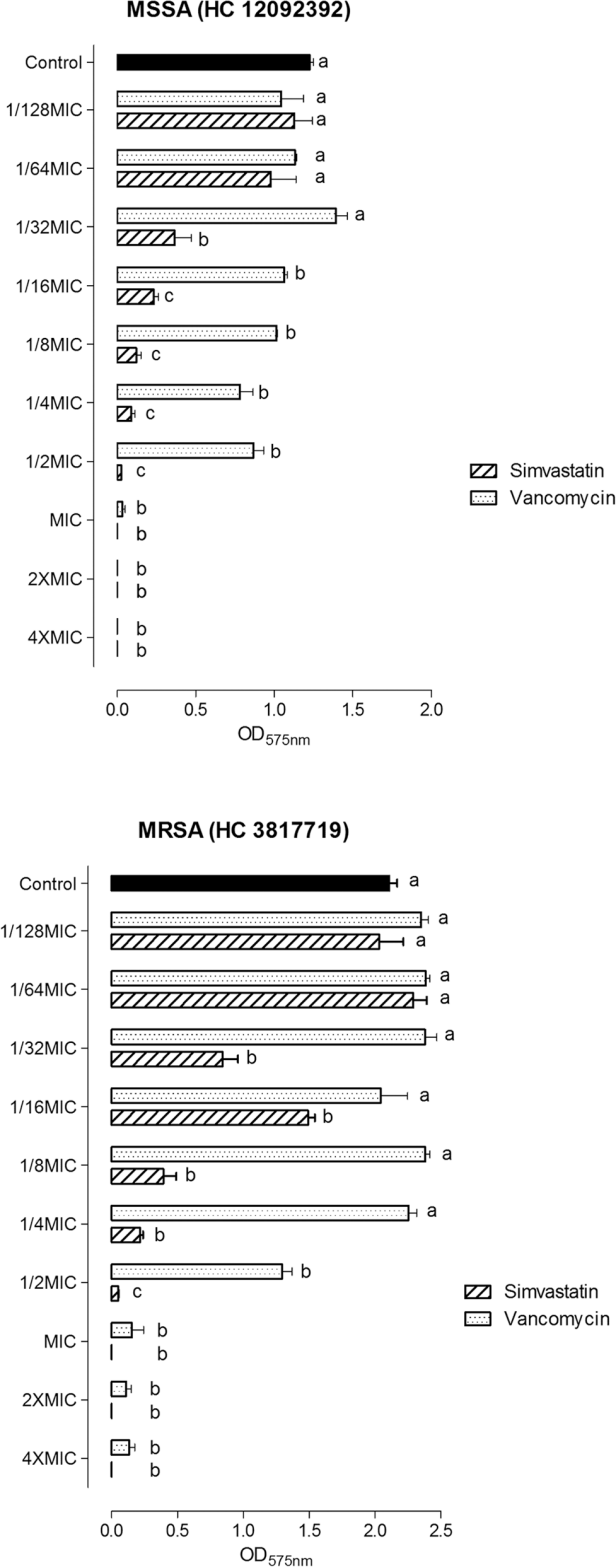
Mean and standard deviation of optical densities (575 nm) representing the biofilm formation of MSSA (HC 12092392) and MRSA (HC 3817719) in the presence of simvastatin and vancomycin. Different letters represent significant differences; comparisons were made for each dilution, the control group and each concentration of simvastatin and vancomycin. (p<0.05, two-way ANOVA, Bonferroni).

Simvastatin in concentrations from 1/16xMIC up to 4xMIC (from 0.98 to 62.6 μg/mL) significantly reduced *S*. *aureus* 29213 biofilm formation (p < 0.05, ANOVA- Bonferroni). A significant inhibition of biofilms was also observed for the antimicrobial standard drug at concentrations higher than 1/2xMIC (from 0.78 to 6.24 μg/mL). When analyzed by MIC range, simvastatin was able to reduce biofilm formation more significantly up to the 1/16xMIC concentration compared to vancomycin (p < 0.05, two-way ANOVA- Bonferroni). For *S*. *aureus* 29213, complete inhibition could be accomplished with simvastatin at 2x and 4xMIC, while this could be accomplished with vancomycin at ½ MIC and MIC concentrations. The images obtained by SEM confirmed the inhibitory effect of simvastatin on *S*. *aureus* 29213 biofilms, showing a reduction in the number of cells and the extracellular matrix of the biofilm (Fig 3B). Simvastatin was also able to reduce biofilm formation of clinical isolates in concentrations from 1/32xMIC up to 4xMIC, while vancomycin reduced bacterial adhesion at concentrations higher than 1/16xMIC for MSSA and 1/2xMIC for MRSA (Fig 4). DMSO did not alter *S*. *aureus* biofilm formation (p > 0.05).

### Simvastatin decreases cell viability and alters the production of polysaccharides in mature biofilms

**Fig 5:**
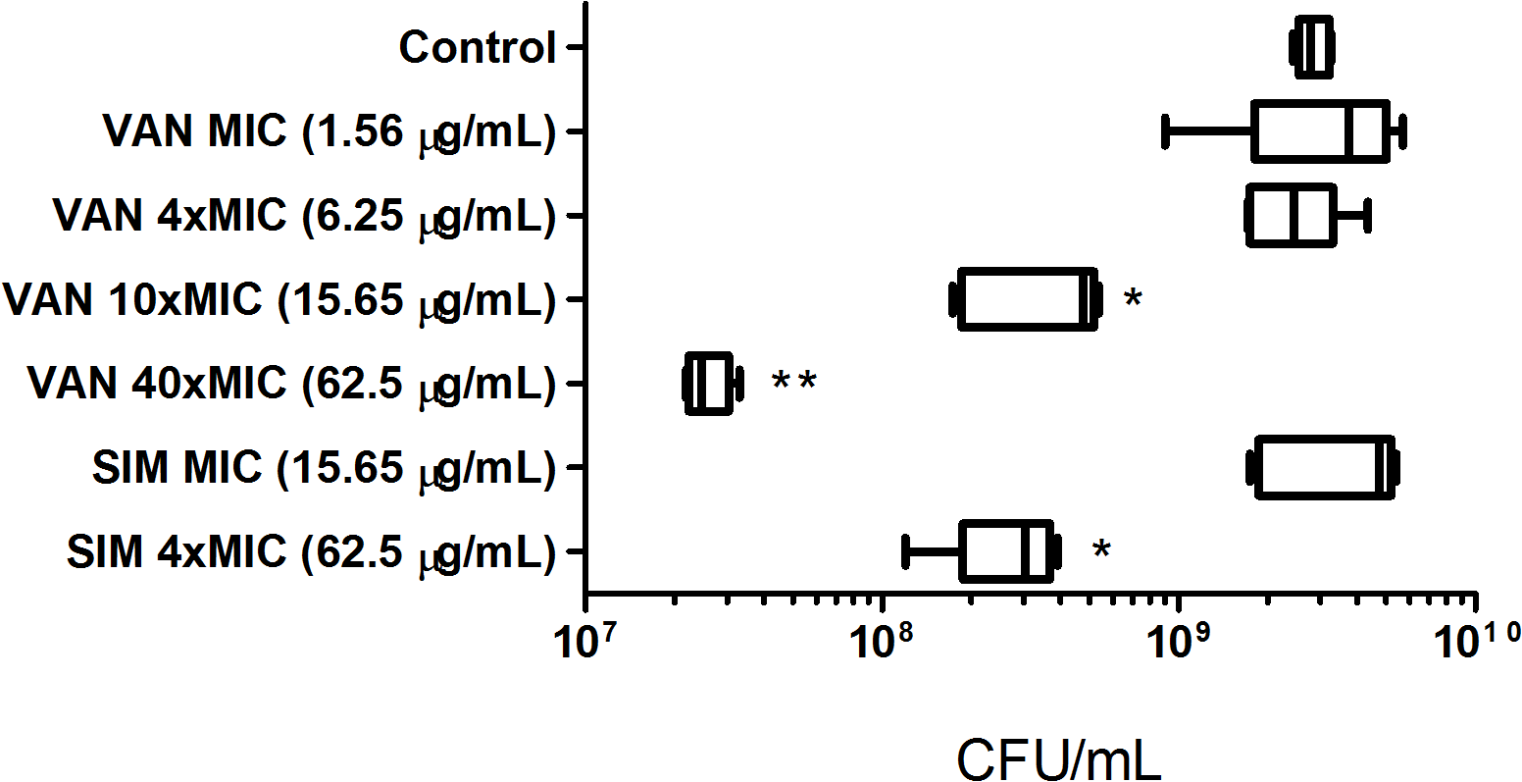
Median and 5–95 percentiles of CFU/mL representing the cell viability of *S*. *aureus* 29213 biofilm after exposure to simvastatin and vancomycin for 24 h. Significant differences between the treatment and the control group when **p < 0.01 (Kruskal-Wallis—Dunn).

In these experiments, after 24 hours of biofilm growth (mature biofilm), *S*. *aureus* biofilms were exposed to simvastatin and vancomycin and viability, polysaccharide and protein production were evaluated. The cell viability of *S*. *aureus* 29213 biofilms after treatment with simvastatin and vancomycin is shown in Fig 5.

The results showed that simvastatin at4xMIC (62.5 μg/mL) was able to significantly reduce viable cells in biofilm when compared to the control and vehicle groups (p<0.005, Kruskal-Wallis, Dunn), but vancomycin at 4xMIC (6.25 μg/mL) showed no difference when compared to the control group (p > 0.05). In concentrations higher than 4xMIC (10xMIC = 15.6 μg/mL and 40xMIC = 62.5 μg/mL), vancomycin also significantly reduced biofilm viability (p > 0.05). The concentration of 62.5 μg/mL Simvastatin was as effective as 15.6 μg/mL vancomycin for killing biofilm. Vancomycin at 62.5 μg/mL showed a more pronounced reduction than simvastatin at 62.5 μg/mL and vancomycin at 15.6 μg/mL (p > 0.05), but did not produce a 100% killing effect. Due to poor solubility, 4xMIC was chosen as the highest concentration tested for simvastatin. DMSO did not reduce the number of viable cells. Thus, simvastatin was effective in reducing bacterial viability of mature *S*. *aureus* 29213 biofilms in a comparable manner to vancomycin.

As simvastatin inhibited *S*. *aureus* ATCC 29213 biofilms, it was hypothesized that this statin could also interfere with the extracellular matrix of the biofilm. The results for insoluble extracellular polysaccharide (EPSI) and intracellular polysaccharide (IPS) are both shown in Table 3. Simvastatin reduced the production of EPSI (p<0.05) and increased the production of IPS when compared with control (p<0.05, Kruskal-Wallis, Dunn). The production of soluble extracellular polysaccharide (EPS) was under the limit of detection. However, simvastatin did not change total protein production (p>0.05; Kruskal-Wallis, Dunn). DMSO did not alter the amount of polysaccharide, protein or biomass.

**Table 3:** Effects of simvastatin on the production of polysaccharides, proteins and biomass of *S*. *aureus* biofilm.

	EPSI (μg/mg)	IPS (μg/mg)	Proteins (μg/mg dry weight)	Biomass (mg)
**Simvastatin**	22.7 ± 9.0^*^	78.6 ± 25.4^*^	2.3 ± 0.4	0.66 ± 0.12^*^
**Control**	40.7 ± 9.6	34.1 ± 8.6	1.9 ± 0.4	0.93 ± 0.13

EPSI- insoluble extracellular polysaccharide. IPS- intracellular polysaccharide. The values of polysaccharides and proteins were normalized by dry weight. Significant differences between the treatment and the control group when:

*p < 0.05 (Kruskal-Wallis).

## Discussion

The potential for the pleiotropic effects of statins has generated studies to investigate the role of these drugs in the prevention, morbidity and mortality of different infections [10–15]. Their antimicrobial activity was previously proposed and investigated [16–19, 21, 22]. We evaluated the antimicrobial activity of atorvastatin, pravastatin and simvastatin against 15 bacterial strains associated with nosocomial infections. In addition, as simvastatin showed activity against *S*. *aureus*, we have explored its effects on *S*. *aureus* planktonic cells and biofilm.

In our study, simvastatin showed 100% inhibition only against *S*. *aureus*. In addition, sub-MIC concentrations were able to reduce the growth of *S*. *aureus* even at concentrations lower than the MIC. The MICs found in the present study were lower than the MICs shown previously in other studies of *S*. *aureus* [19] and other bacterial species [22]. However, the values found for antimicrobial standards are in accordance with CLSI [27], demonstrating that the method used in our study was suitable. Finally, Bergman et al. [18] found a MIC value of 15.6 μg/mL for simvastatin against *Streptococcus pneumoniae*, which is a concentration that is very close to the one found in the present study.

Atorvastatin and pravastatin did not present full inhibitory activity, as demonstrated by the MIC tests. However, pravastatin significantly reduced the growth of *S*. *aureus*. In the present study, it was also observed that simvastatin and pravastatin had a slightly inhibitory effect on growth in *E*. *coli*, *P*. *aeruginosa* and *E*. *faecalis* (data not shown). Masadeh et al. [22] previously reported inhibitory activity for simvastatin and atorvastatin against several species, including *E*. *coli*, *P*. *aeruginosa* and *E*. *faecalis*. The enhanced antimicrobial activity of simvastatin in comparison to pravastatin and atorvastatin may be related to differences in their chemical characteristics, as described previously [19, 22]. Pravastatin and simvastatin are semi-synthetic forms that are derivatives of lovastatin, a metabolic product of *Penicillium citrinum* [38], and atorvastatin is the pure synthetic form. Simvastatin and atorvastatin are lipophilic, but pravastatin has hydrophilic properties [39]. Thus, simvastatin probably crosses the cell membrane more easily, causing bacterial inhibition in a dose-dependent manner. Although lipophilic, atorvastatin has no significant antimicrobial activity. This molecule is not derived from a fungal metabolite, which may be the reason for lacking antimicrobial effects. However, further studies on the structure-activity relationship should be conducted to better understand the antimicrobial properties of statins.

To better understand the antimicrobial properties of statins, we investigated their effects on *S*. *aureus* 29213 in planktonic and biofilm assays. We first evaluated cell viability when *S*. *aureus* 29213 was exposed to simvastatin for 2, 4, 8 and 12 h (time-kill assays). At 4xMIC, simvastatin reduced the number of viable cells, especially after 12 h of exposure. However, at the MIC, the number of viable cells remained constant during all periods of exposure. Vancomycin showed a bactericidal effect at both concentrations and reduced the number of viable cells more significantly than simvastatin. In addition, we also verified how long these effects persist after removal of the drug, also known as the post-antibiotic effect (PAE). For the 4xMIC concentration, simvastatin and vancomycin showed a similar PAE. In this experiment, bacteria was exposed to the antibiotics for a short period (2 h) and then transferred to a drug free medium. Simvastatin and vancomycin showed a suppressive effect on *S*. *aureus* growth that persisted after drug removal, in the “recovery period”. The ability of simvastatin to produce a PAE similar to vancomycin is an interesting finding, as theoretically it could suppress bacterial growth for a longer period of time, even when concentrations fall below the MIC [30]. PAE is an important property that impacts on antimicrobial regimens, as substances that do not present this effect require more frequent antimicrobial administration than those that present this post-effect [30].

Previous studies have reported *in vitro* synergistic and antagonistic effects of the association between statins and antifungals [40–42]. Therefore, we investigated a possible interaction between simvastatin and vancomycin using a checkerboard test. However, the combination of these drugs had no synergistic effect against any strain of *S*. *aureus*. The FICI value found for *S*. *aureus* 29213 was low compared to the other strains, but more studies are needed to verify if this interaction has some potential.

Despite the importance of determining the MIC and antibacterial activity against planktonic cells, microorganisms are not usually found in body liquids [43]. To face the adversities of the environment, microorganisms adhere to surfaces and grow grouped together with extracellular polymeric substances (EPS), forming communities known as biofilms [43, 44]. Biofilm formation involves four steps: (1) reversible adhesion to a surface, (2) production of EPS (irreversible adhesion), (3) microcolony formation and intense production of EPS and (4) biofilm dispersion [44, 45].

Our results show that simvastatin was able to inhibit biofilm formation in concentrations 16 times lower than MIC for *S*. *aureus* 29213 and 32 times lower than MIC for clinical isolates. This inhibition was confirmed by images obtained by scanning electron microscopy (SEM). Considering both vehicle and control groups, it was possible to observe a biofilm at a mature stage with the cells immersed in an extracellular matrix. In the presence of simvastatin, however, few cells adhered to the surface with drug concentrations equal to or higher than the MIC. For the ½xMIC concentration, more cells adhered, but the biofilm failed to develop and achieve step 3. Thereby, simvastatin inhibits biofilm formation by *S*. *aureus*, probably preventing the adherence of cells at concentrations higher than the MIC and the development of biofilms at sub-MIC concentrations.

The fight against mature biofilms has become a challenge. They are more resistant to antimicrobial agents, which makes treatment difficult and leads to complications for the patient [46]. We investigated the effect of simvastatin on *S*. *aureus* biofilms with 24 h of growth at a more advanced stage of maturation. Simvastatin exhibited considerable activity at 4xMIC (62.5 μg/mL), reducing cell viability with similar effects to vancomycin 10xMIC (15.6 μg/mL) In terms of concentration in μg/mL, vancomycin showed killing effects in smaller concentrations than simvastatin. However, when comparing both substances in terms of MIC range, simvastatin showed better results. The concentration of antibiotic required to kill cells in biofilms is much higher than to kill planktonic cells, sometimes 100 or 1000 times the MIC [26]. Therefore, our findings revealed a potential for simvastatin to be explored because in concentrations only 4 times the MIC it has the ability to decrease cell viability. Unfortunately, the difficulty of diluting simvastatin did not allow testing at concentrations higher than 4xMIC for biofilm tests

Several mechanisms explaining the resistance of biofilms have been described [26, 46]. The extracellular matrix is implicated as an important mechanism, especially in decreasing the penetration of an antibiotic [26]. We hypothesized that the effect of simvastatin could also involve an effect on two important components of EPS, polysaccharides and proteins. The EPSI contributes to structure and is responsible for the integrity of biofilms [43]. Some EPSI are also associated with resistance in bacterial biofilms. For example, poly-(1,6)-*N*-acetyl-D-glucosamine (PNAG), the major extracellular polysaccharide in *S*. *aureus*, is responsible for preventing fluid convection and the transport of solute through biofilms [47]. After treatment with simvastatin, the biofilm showed a reduction in the production of EPSI and an increase in the production of IPS when compared to the control group. The increase in production of IPS in some bacteria, such as *S*. *mutans*, is associated with a nutrition reservoir allowing the extension of survival in limiting conditions [48]. A possible hypothesis for this finding would be that in an attempt to increase its survival in the presence of simvastatin, *S*. *aureus* decreases the production of EPSI to produce reserve polysaccharides. This change could also explain the excellent effect of simvastatin on *S*. *aureus* viability. Because EPSI is responsible for biofilm structure and is a major component of the extracellular matrix, a reduction in its production could lead biofilms to be more accessible to drugs. However, more studies are needed to understand the role of simvastatin in the production of polysaccharides.

Simvastatin did not significantly alter the amount of protein. The production of proteins is generally more intense during biofilm formation, because they play a key role in the colonization of biofilms [44]. Thus, the absence of an effect on protein production is understandable. Perhaps a qualitative study can better determine if simvastatin has some effect on the production of proteins. The reduction in the biomass of biofilms is probably due to a reduction in polysaccharides because they are the major fraction of the EPS [44].

The concentrations found to have antimicrobial properties are a thousand times higher than the plasmatic concentrations achieved in patients undergoing statin therapy [18]. Thus, simvastatin doses have to be increased for clinical purposes, but safety in higher doses is unknown. In addition, it is unknown if the antimicrobial concentrations found in the present study would be achievable in plasma considering its poor solubility. However, regardless of whether the physiological concentrations of simvastatin inhibit bacterial activity, our results highlight an antimicrobial potential to be explored. Our study was the first to investigate the effect of simvastatin on bacterial biofilms, showing a substantial antimicrobial activity for this statin. The identification and development of new antibiotics, especially those with new mechanisms of action, are of utmost importance for public health worldwide [49]. As other antibiotics, simvastatin would serve in future studies as a synthetic scaffold to originate new antibiotics. For example, prontosil, a sulfa class of antibiotics was first developed as a dye and nalidixic acid, was an intermediate in the synthesis of chloroquine (Fischbach1 and Walsh, 2009). We believe that studies of their molecular structure would introduce a new antibacterial pharmacophore, which would be useful in the future as a template for the development of new antibiotics.

In conclusion, simvastatin has antimicrobial activity against *S*. *aureus* biofilms, reducing their formation, viability and polysaccharide production. These findings may contribute to the search for new antibacterial drugs with consideration of the potential for simvastatin as an antibiotic prototype.
